# Expression of Mitophagy-Associated Factors and Their Associations with Metastasis and Prognosis in Oral Squamous Cell Carcinoma

**DOI:** 10.3390/cancers18193194

**Published:** 2026-10-03

**Authors:** Yudai Matsuzoe, Daisuke Takeda, Izumi Saito, Yuki Murakami, Aki Murakami, Yoshiaki Tadokoro, Junya Hirota, Yasumasa Kakei, Masaya Akashi, Takumi Hasegawa

**Affiliations:** Department of Oral and Maxillofacial Surgery, Kobe University Graduate School of Medicine, Kobe 650-0017, Japan; 243m913m@stu.kobe-u.ac.jp (Y.M.); izumi.kurosawa@kohnan.or.jp (I.S.); 268m910m@gsuite.kobe-u.ac.jp (Y.M.); aki0221@med.kobe-u.ac.jp (A.M.); tadokoro-yoshiaki@med.kobe-u.ac.jp (Y.T.); junjun_trade_art@yahoo.co.jp (J.H.); ykakei@med.kobe-u.ac.jp (Y.K.); akashim@med.kobe-u.ac.jp (M.A.); takumi0531h@ybb.ne.jp (T.H.)

**Keywords:** squamous cell carcinoma, TFEB, NRF2, ATF4, Parkin, PINK1, MITOL (MARCH5)

## Abstract

Background/Objectives: Mitophagy-associated factors have been implicated in tumor behavior and patient outcomes; however, their clinical significance depends on tumor type, stage, and microenvironmental context, and consistent conclusions remain lacking. We aimed to comprehensively assess the expression of mitophagy-associated factors in a moderate-sized cohort of patients with oral squamous cell carcinoma (OSCC) and clarify their prognostic significance. Methods: A retrospective cohort study included 197 patients with OSCC who underwent primary surgical resection between August 2013 and October 2018. Immunohistochemical expression of mitophagy-associated factors, including transcription factor EB (TFEB), nuclear factor erythroid 2-related factor 2 (NRF2), activating transcription factor 4 (ATF4), Parkin, PTEN-induced kinase 1 (PINK1), and mitochondrial ubiquitin ligase (MITOL), was evaluated. Associations between protein expression and clinicopathological parameters, local control, 5-year disease-specific survival (DSS), regional control, and distant metastasis were analyzed. Results: High expression of NRF2 and TFEB was significantly related with decreased DSS, poor regional control, and increased distant metastasis. High ATF4 expression was associated with distant metastasis and showed no association with DSS. Parkin, PINK1, and MITOL showed no significant association with patient outcomes. Explanatory multivariable analysis showed associations between high TFEB expression, vascular invasion, extranodal extension, and disease-specific mortality. Conclusions: TFEB expression showed an exploratory association with aggressive tumor behavior and poor prognosis in OSCC, warranting further investigation. However, because immunohistochemical evaluation measures protein expression rather than mitophagy flux or mitochondrial turnover, these findings represent associations between mitophagy-associated factor expression and clinical outcomes and do not directly demonstrate altered mitophagy activity.

## Simple Summary

Mitophagy is a cellular process that removes damaged mitochondria and helps maintain normal cell function. Although abnormalities in mitophagy have been implicated in cancer progression, their clinical significance in oral squamous cell carcinoma (OSCC) remains unclear. In this retrospective study, we evaluated the expression of six mitophagy-associated factors (NRF2, TFEB, Parkin, PINK1, MITOL, and ATF4) in surgically treated patients with OSCC and examined their relations with clinicopathological features and prognosis. High expression of NRF2 and TFEB was significantly associated with poor disease-specific survival, worse regional control, and increased risk of distant metastasis, whereas high ATF4 expression was associated with distant metastasis. Parkin, PINK1, and MITOL showed no significant prognostic value. Explanatory multivariable analysis showed an association between high TFEB expression and disease-specific mortality. These findings suggest an exploratory association between TFEB expression and prognosis in patients with OSCC, although its biological role and association with mitophagy activity require further investigation.

## 1. Introduction

Mitophagy is a selective form of autophagy responsible for the removal of damaged mitochondria and plays an essential role in maintaining mitochondrial quality control and cellular homeostasis [1,2,3]. This process is tightly regulated by multiple molecular factors, among which transcription factor EB (TFEB), nuclear factor erythroid 2-related factor 2 (NRF2), activating transcription factor 4 (ATF4), Parkin, PTEN-induced kinase 1 (PINK1), and mitochondrial ubiquitin ligase (MITOL; also known as membrane-associated ring-CH-type finger 5 [MARCH5]) have been identified as key regulators of mitophagy.

These six factors were selected to represent distinct components and regulatory mechanisms associated with mitophagy and mitochondrial quality control, including the PINK1–Parkin pathway and broader transcriptional stress responses.

The autophagy–lysosome system, including mitophagy, is orchestrated by the master transcription factor TFEB [4]. In normal cells, TFEB promotes the clearance of unnecessary or damaged intracellular components and exerts cytoprotective functions. However, recent studies have reported that constitutive activation of TFEB in tumor cells does not necessarily act in a tumor-suppressive manner and may instead support tumor progression.

The PINK1/Parkin pathway represents a major mitophagy mechanism that selectively recognizes and eliminates damaged mitochondria [1,2]. Impaired function of PINK1 or Parkin leads to reactive oxygen species (ROS) accumulation and mitochondrial dysfunction, a process linked to tumor progression and malignant transformation [1,2,3]. MITOL suppresses excessive mitophagy by promoting the degradation of phosphorylated Parkin, and loss of MITOL function has been associated with mitochondrial impairment, cell death, and potential tumor progression [5].

NRF2 and ATF4 are stress-responsive transcription factors activated under oxidative or metabolic stress, and both demonstrate functional connections to mitophagy. NRF2 contributes to antioxidant defense and mitochondrial quality control in normal cells, and adequate NRF2 activity may be associated with favorable outcomes [6]. In contrast, constitutive NRF2 activation promotes treatment resistance and tumor progression in advanced cancers [6,7].

ATF4 is a central regulator of the integrated stress response and supports cellular adaptation to mitochondrial and oxidative stress. Although ATF4 activation enhances antioxidant capacity and metabolic regulation, excessive or sustained ATF4 signaling has been linked to tumor invasion, metastasis, and therapeutic resistance [8].

Mitophagy demonstrates a dual role in cancer biology. In contexts characterized by physiological mitochondrial quality control, enhanced mitophagy activity or upregulation of associated factors has often been linked to a favorable prognosis. Conversely, in advanced tumors, these pathways may be co-opted by cancer cells to support survival, adaptation, and progression, ultimately functioning as adverse prognostic factors. Although mitophagy-associated factors have been implicated in tumor behavior and patient outcomes, their clinical significance appears to depend strongly on tumor type, stage, and microenvironmental context, and consistent conclusions remain lacking. In oral squamous cell carcinoma (OSCC), individual factors such as TFEB, NRF2, ATF4, Parkin, PINK1, and MITOL have been investigated, although findings remain inconsistent across studies. Moreover, no prior work has comprehensively evaluated multiple mitophagy-associated factors in a large OSCC cohort while examining their relationships with clinicopathological characteristics and prognosis. Thus, mitophagy represents a highly controversial field. In addition, in our previous study, Murakami et al. reported associations between five mitochondrial-related factors, PGC-1α, TFAM, OGG1, MTUS1, and SIRT3, and tumor differentiation, metastasis, and prognosis in OSCC [9]. Building on these findings, in the present study, we focused on mitophagy-associated factors, which represent another important aspect of mitochondrial quality control, and we investigated their associations with tumor progression and prognosis.

Therefore, in this study, we aimed to comprehensively assess the expression of mitophagy-associated factors in a moderate-sized cohort of patients with OSCC and to clarify their prognostic significance. We hypothesized that differences in the expression of these mitophagy-associated factors would be associated with clinicopathological characteristics and patient outcomes. Therefore, immunohistochemical analyses and clinicopathological evaluations of mitophagy-associated regulatory factors were performed.

## 2. Materials and Methods

A total of 197 patients diagnosed with OSCC who underwent surgical resection at the Department of Oral and Maxillofacial Surgery, Kobe University Hospital (Kobe, Japan), between August 2013 and October 2018 were included in this study [9]. Clinical, pathological, and epidemiological information was retrospectively collected from medical records. Clinicopathological data analyzed included age, sex, smoking history, alcohol use, performance status (PS), tumor subsite, clinical T classification based on the 8th edition of the Union for International Cancer Control/American Joint Committee on Cancer staging system, histological characteristics (e.g., tumor differentiation, vascular, lymphatic, and perineural invasion), pathological lymph node findings (including extranodal extension [ENE] and multiple lymph node metastases [MLM]), and treatment outcomes.

All patients were managed in accordance with established clinical guidelines for oral cancer [10]. Only patients with histologically confirmed OSCC who underwent curative surgery were included. Individuals who received preoperative chemotherapy and/or radiotherapy were excluded from the study. No patients were excluded from the principal analyses because of missing tissue samples or unavailable clinical data.

Surgical margin status was classified according to the National Comprehensive Cancer Network criteria [11]. A positive margin was defined as an invasive tumor present at the inked surgical margin, whereas a close margin was defined as a tumor-to-margin distance of <5 mm.

This study was approved by the Ethics Committee of Kobe University Hospital (approval number: B250114). As this was a retrospective analysis, informed consent was not mandatory; however, all patients were provided the option to opt out of participation regarding the use of their surgical specimens.

Immunohistochemical analysis was conducted using 4 µm thick sections obtained from formalin-fixed, paraffin-embedded tumor tissues prepared in the pathology laboratory. Sections were deparaffinized in xylene, rehydrated through graded ethanol solutions, and rinsed three times with phosphate-buffered saline. Antigen retrieval was achieved by heat treatment in an ethylenediaminetetraacetic acid buffer (pH 9.0; Dako, Carpinteria, CA, USA) using a water bath for 30 min. To block endogenous peroxidase activity, sections were incubated with 3% hydrogen peroxide. Tissue sections were then incubated overnight at 4 °C with appropriately diluted primary antibodies prepared in Can Get Signal Immunostain Solution A (Toyobo, Osaka, Japan): rabbit polyclonal anti-NRF2 (1:150; bs–2013R; Bioss Antibodies, Woburn, MA, USA), rabbit polyclonal anti-TFEB (1:200; 13372–1–AP; Proteintech, Rosemont, IL, USA), rabbit polyclonal anti-Parkin (PARK2) (1:200; 14060–1–AP; Proteintech), rabbit polyclonal anti-PINK1 (1:100; 23274–1–AP; Proteintech), rabbit polyclonal anti-MITOL (MARCH5) (1:100; 12213–1–AP; Proteintech), and rabbit polyclonal anti-ATF4 (1:200; 10835–1–AP; Proteintech).

Subsequently, sections were incubated for 1 h at room temperature (22–25 °C) with an undiluted horseradish peroxidase-conjugated anti-rabbit immunoglobulin G polyclonal antibody (#424142; Nichirei Bioscience, Tokyo, Japan). Immunoreactivity was visualized as a brown precipitate following incubation with the peroxidase substrate 3,3-diaminobenzidine (#415171; Nichirei Bioscience) for 10 min at room temperature. Sections were then counterstained with hematoxylin and examined at ×200 magnification using a BZ-X800 microscope (Keyence, Osaka, Japan). Protein expression levels were jointly evaluated and quantified by two observers using the BZ-H3C/Hybrid Cell Count software (Keyence, Osaka, Japan). Positive and total cell numbers were quantified using the automated color-recognition algorithm of the BZ-H3C/Hybrid Cell Count software, which identifies immunopositive cells based on staining color/hue rather than on a manually defined intensity threshold. Tumor epithelial cells were identified and segmented visually by the observers based on morphology and were distinguished from stromal and inflammatory cells before quantification of positive and total cell counts within the selected tumor areas. Staining was evaluated predominantly in the cytoplasm; cells showing nuclear staining were also included as positive. The two observers, who were blinded to the clinical outcomes, assessed the cells together and reached consensus in real time for each field, and the resulting consensus measurements were used to construct the final dataset. Tumor regions were evaluated at the invasive front, which was selected as the region of interest for all cases. Necrotic areas were excluded from the analysis. Within the invasive front, three microscopic fields per case were randomly selected at ×200 magnification for cell counting, without reference to staining intensity. Each field measured approximately 1.06 mm × 0.79 mm, corresponding to an analyzed area of approximately 0.84 mm^2^ per field and approximately 2.52 mm^2^ per case across three fields. Negative control sections without primary antibodies were included in each staining batch to assess nonspecific staining. A common reference tissue was included in all batches to monitor batch-to-batch consistency, and no major variation in staining intensity was observed. Dedicated positive control tissues known to strongly express each target protein were not included in the original staining protocol.

The expression status of the six proteins was dichotomized into positive and negative groups using the median value as the cutoff for each marker. This median-based approach ensured an unbiased and reproducible classification of protein expression levels across the cohort. Accordingly, cases with expression levels greater than the median were classified as positive (high expression), whereas those with expression levels equal to or below the median were classified as negative (low expression). The median cutoff values for NRF2, TFEB, Parkin, PINK1, MITOL, and ATF4 were 47.57%, 38.20%, 51.54%, 50.13%, 38.71%, and 52.84%, respectively (Table 1).

Cumulative local control (LC), regional control (RC), distant metastasis (DM), and 5-year disease-specific survival (DSS) rates were estimated using the Kaplan–Meier product-limit method. LC, RC, and DM control were defined based on the occurrence of local, regional, and distant recurrence, respectively, and were calculated from the date of surgery to the date of the first corresponding event or to the last follow-up. These endpoints were not mutually exclusive; 38 patients (19.3%) experienced more than one type of event during follow-up. DSS was defined as the interval from the date of surgery to the date of OSCC-specific death, with deaths from other causes censored at the time of death. The median follow-up period was 85.0 months (95% confidence interval [CI], 82–91), calculated using the reverse Kaplan–Meier method, with a data cutoff date of 28 October 2025.

The relationship between operative variables, including the expression status of the six proteins, and DSS was examined using multivariable Cox proportional hazards models. In addition, to verify the previous and present study, we examined a total of 11 variables, including five mitochondrial-related factors, in the explanatory multivariable analysis. A forward stepwise selection procedure was applied to eliminate variables that did not significantly contribute to the model. In this analysis, we created an initial model by forcing all clinical factors, except operative variables, into the model. Variables were then added in order of those showing the greatest improvement to the model. Hazard ratios (HRs) and corresponding 95% CIs were calculated.

## 3. Results

The clinicopathological characteristics of patients with OSCC are summarized in Table 2. Among 197 patients included in this study, 106 (53.8%) were male and 91 (46.2%) were female, with a mean age of 67.5 ± 13.0 (range, 15–89) years. The tongue was the most frequent primary tumor site (92 cases, 46.7%), followed by the lower gingiva (43, 21.9%), upper gingiva (30, 15.2%), and buccal mucosa and floor of the mouth (16 each, 8.1%). Regarding tumor classification, 36 (18.3%) patients were classified as T1, 66 (33.5%) as T2, 30 (15.2%) as T3, and 65 (33.0%) as T4a or T4b. Concerning outcomes, 20 (10.2%) patients died owing to local recurrence, 32 (16.2%) owing to regional recurrence, 32 (16.2%) owing to DM, and 33 (16.8%) owing to disease-specific death. Twenty-one (10.7%) patients died from other causes, and 143 (72.6%) were alive at the last follow-up (Table 2).

As a sensitivity analysis, cumulative incidence functions accounting for competing risks were additionally calculated, and the estimates were substantially consistent with those obtained using the standard Kaplan–Meier method.

**Figure 1:**
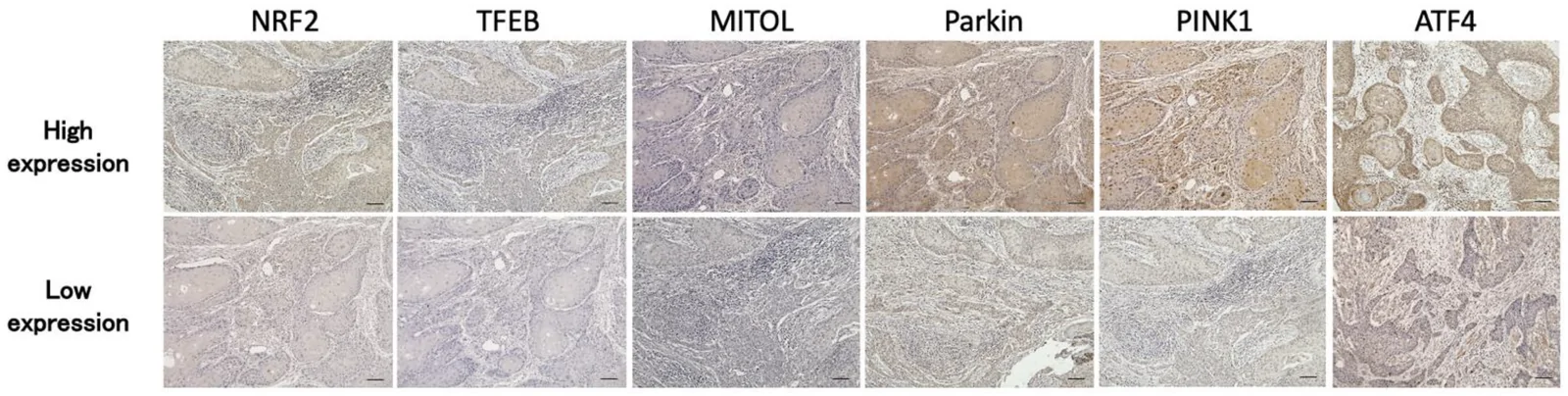
Expression analysis of the six selected proteins using immunohistochemistry. Immunostaining showed that NRF2, TFEB, and ATF4 exhibited significant nuclear staining in the positive group, whereas cytoplasmic staining was observed in the negative group. Parkin, PINK1, and MITOL showed cytoplasmic staining in the positive group. The images are shown at ×200 magnification. Scale bar = 50 μm. NRF2, nuclear factor erythroid 2-related factor 2; TFEB, transcription factor EB; ATF4, activating transcription factor 4; PINK1, PTEN-induced kinase 1; MITOL, mitochondrial ubiquitin ligase.

Immunohistochemical analysis revealed that NRF2, TFEB, and ATF4 exhibited prominent nuclear staining in the positive groups, whereas staining was predominantly cytoplasmic in the negative groups. In contrast, Parkin, PINK1, and MITOL showed cytoplasmic staining in the positive groups. Representative expression patterns of the six proteins are shown in Figure 1. Associations between the expression of these six proteins and clinicopathological parameters were subsequently evaluated (Table 3).

No significant associations were observed between protein expression and age, sex, PS, smoking status, alcohol consumption, lymphatic invasion, vascular invasion, perineural invasion, histological differentiation, or ENE of lymph node metastasis.

Regarding the primary tumor site (tongue vs. non-tongue), MITOL expression showed a significant association (*p* = 0.001). In contrast, no significant associations were observed between the primary tumor site and expression of NRF2, TFEB, Parkin, PINK1, or ATF4.

NRF2 was more frequently negative in advanced-stage cancer than in early-stage cancer (*p* = 0.015), whereas Parkin showed a similar but nonsignificant trend (*p* = 0.064).

For clinical N stage, no significant differences were observed for any of the molecules; however, NRF2, TFEB, and MITOL showed trends toward an association (*p* = 0.059, *p* = 0.059, and *p* = 0.059, respectively). Concerning MLM, only NRF2 showed a marginal trend toward an association (*p* = 0.052).

Regarding surgical margin status, ATF4 expression showed a significant association, with positive cases more frequent in specimens with close or positive margins (*p* = 0.043). No significant associations were observed between the other molecular markers and surgical margin status.

**Figure 2:**
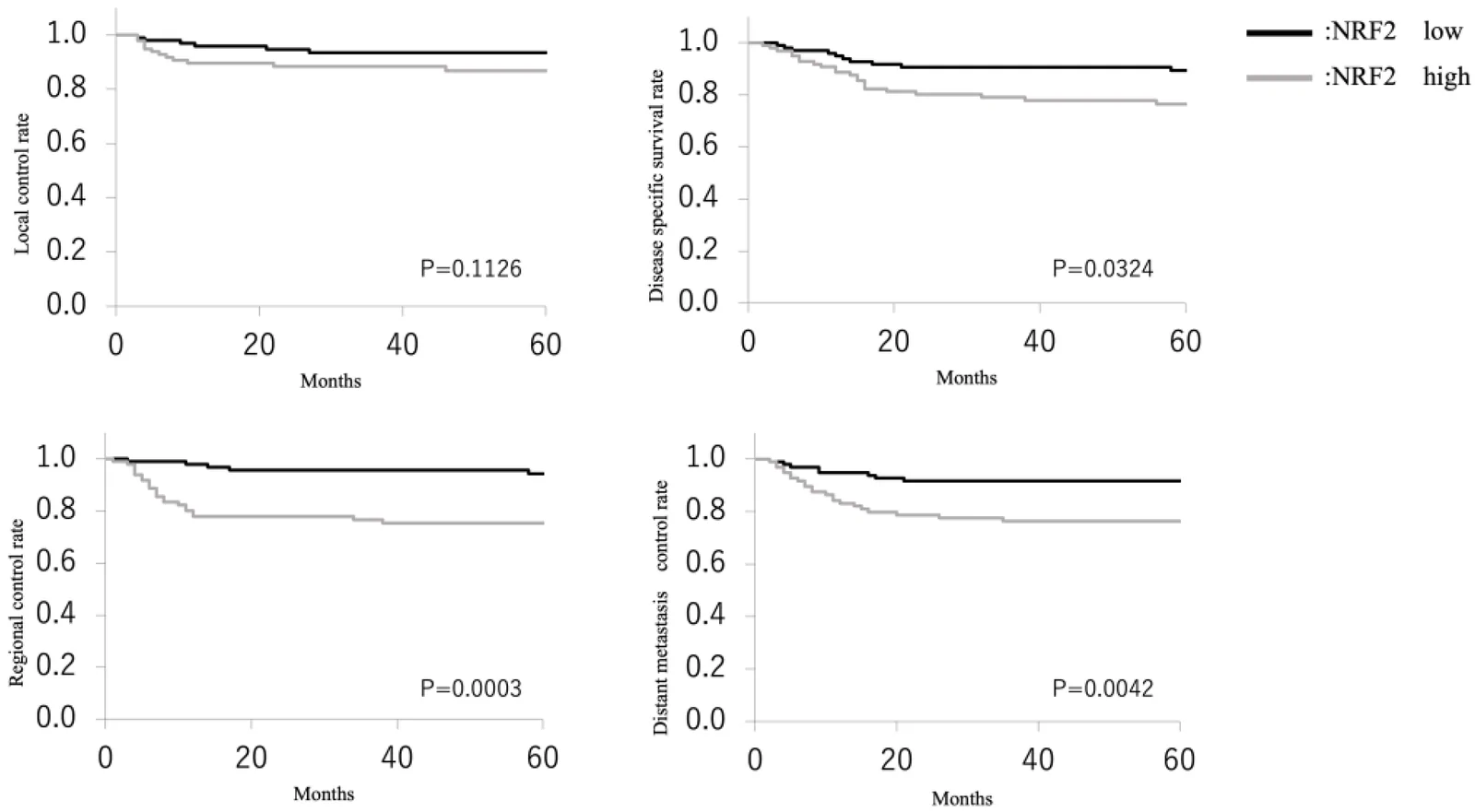
The high NRF2 expression group showed significantly worse DSS than the low expression group (*p* = 0.032). Additionally, the RC (*p* < 0.001) and DM control (*p* = 0.004) rates were significantly decreased. In contrast, no significant difference was observed in the LC rate (*p* = 0.113).

**Figure 3:**
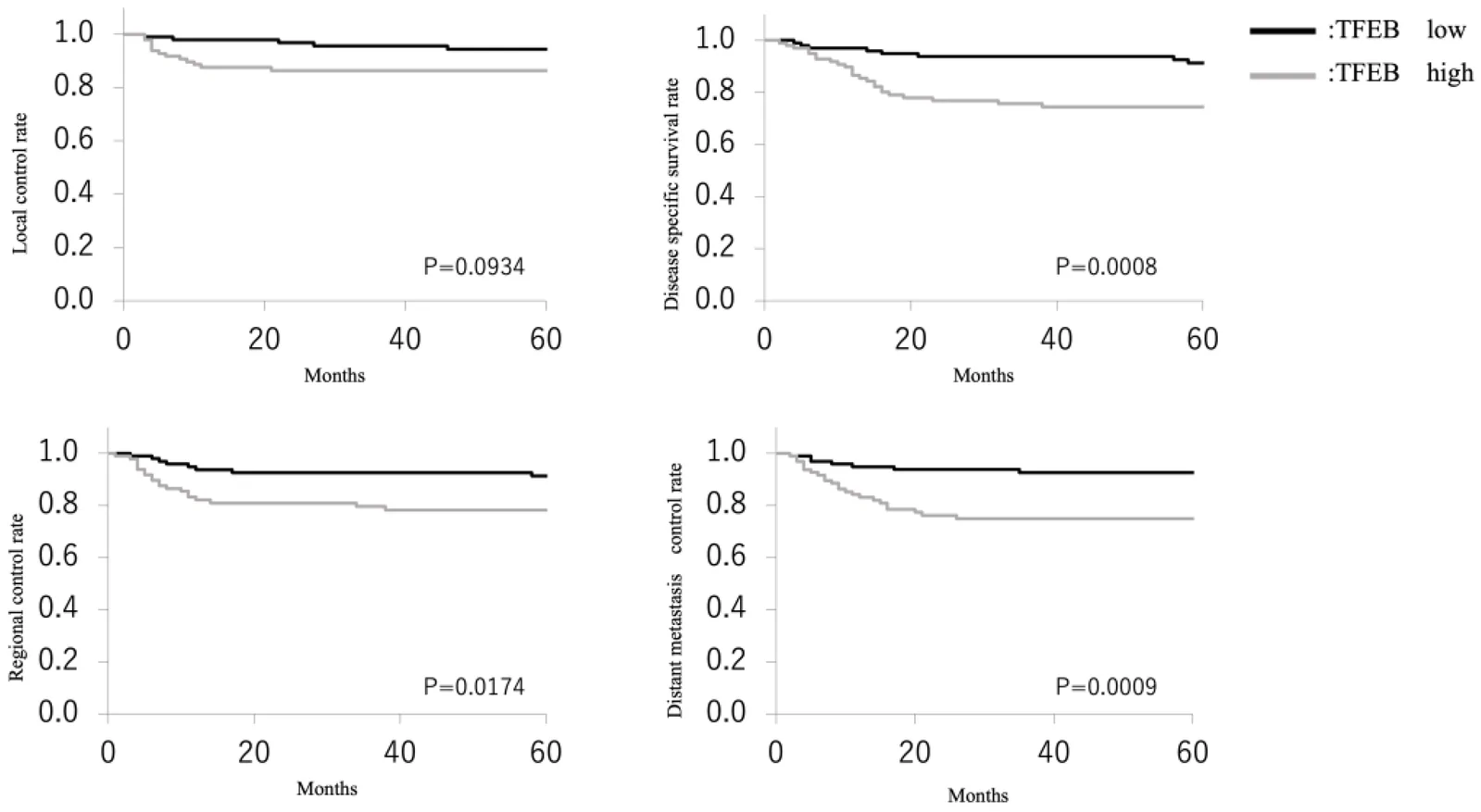
DSS was significantly worse in the high TFEB expression group than in the low expression group (*p* < 0.001). Furthermore, significant decreases were observed in the RC (*p* = 0.017) and DM control (*p* < 0.001) rates. No significant difference was observed in the LC rate (*p* = 0.093).

**Figure 4:**
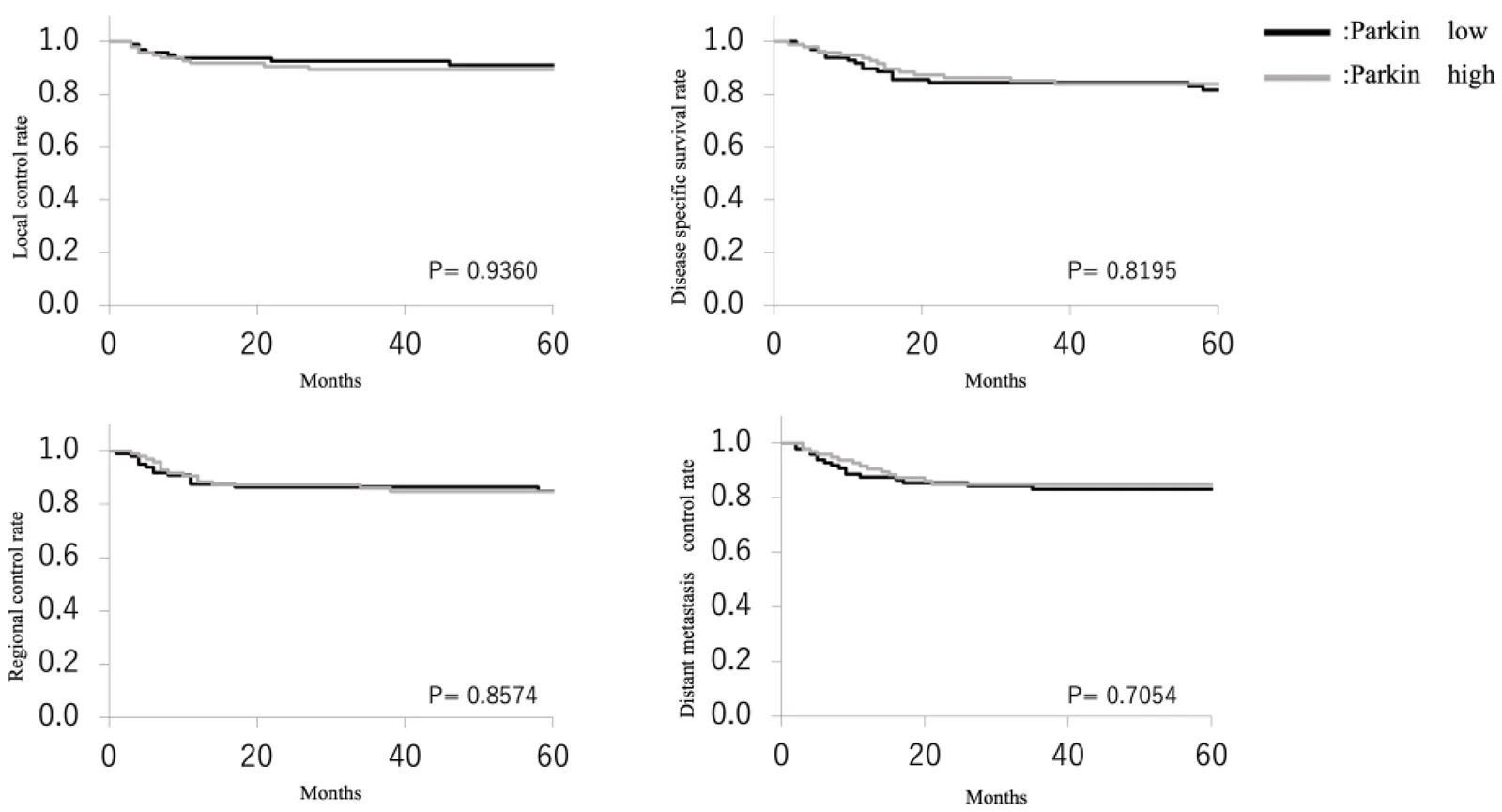
In comparisons based on Parkin expression levels, no significant differences were observed in the LC rate (*p* = 0.936), DSS (*p* = 0.820), RC rate (*p* = 0.857), or DM control rate (*p* = 0.705).

**Figure 5:**
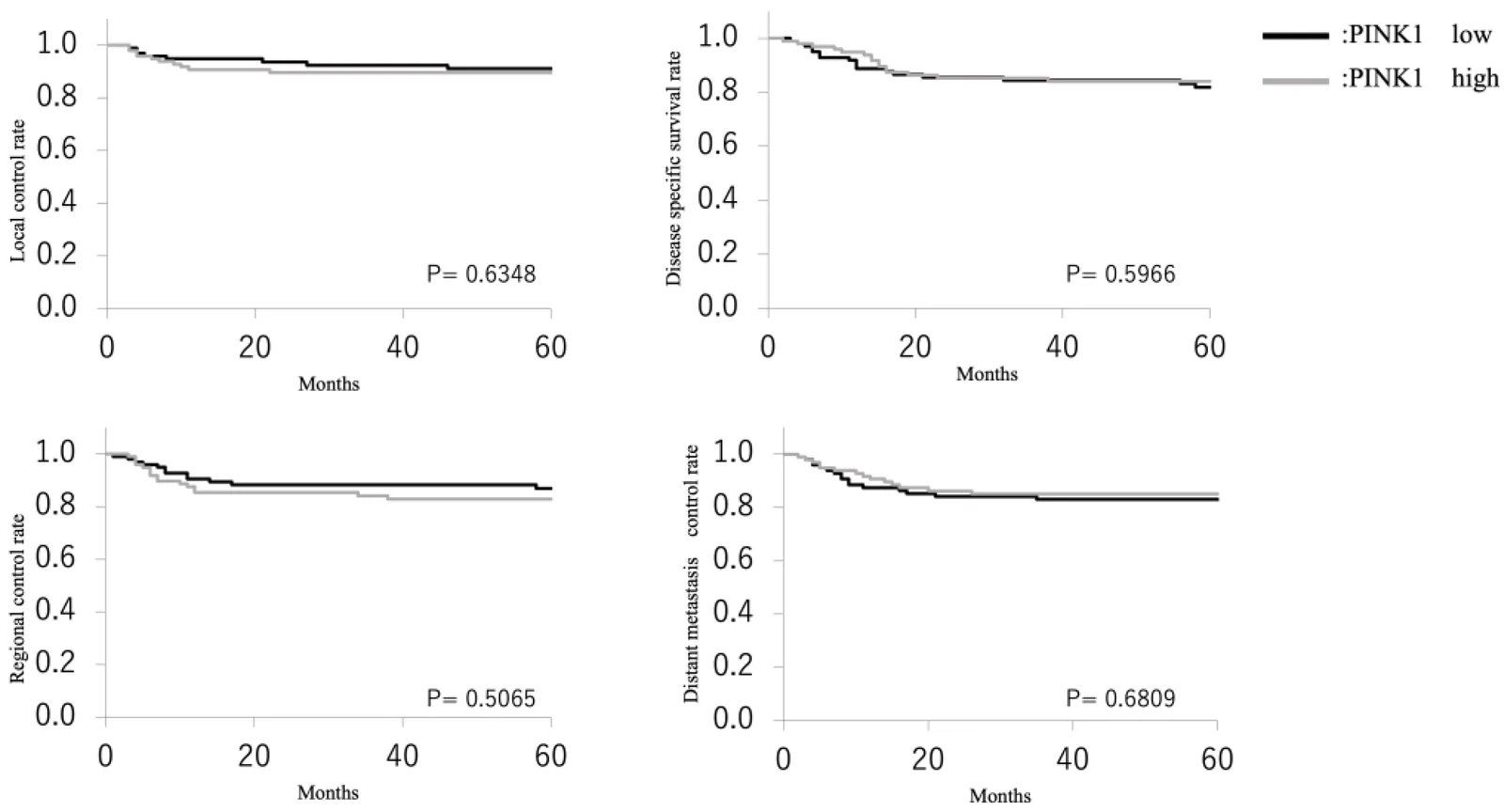
Between the high and low PINK1 expression groups, no significant differences were observed in the LC (*p* = 0.635), DSS (*p* = 0.597), RC (*p* = 0.507), or DM control (*p* = 0.681) rate.

**Figure 6:**
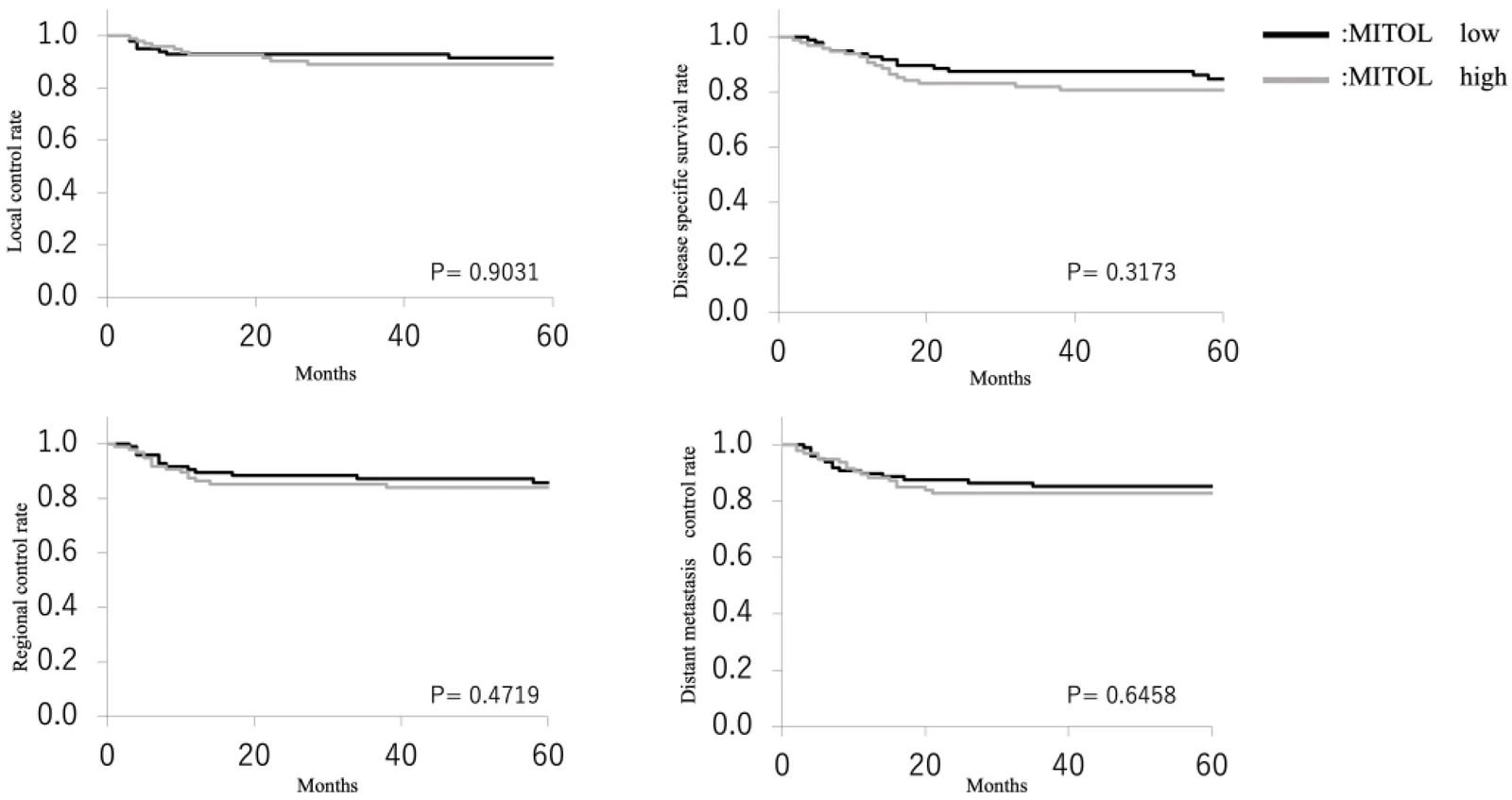
In the analysis of MITOL expression levels, no significant differences were observed in the LC rate (*p* = 0.903), DSS (*p* = 0.317), RC rate (*p* = 0.472), or DM control rate (*p* = 0.646).

**Figure 7:**
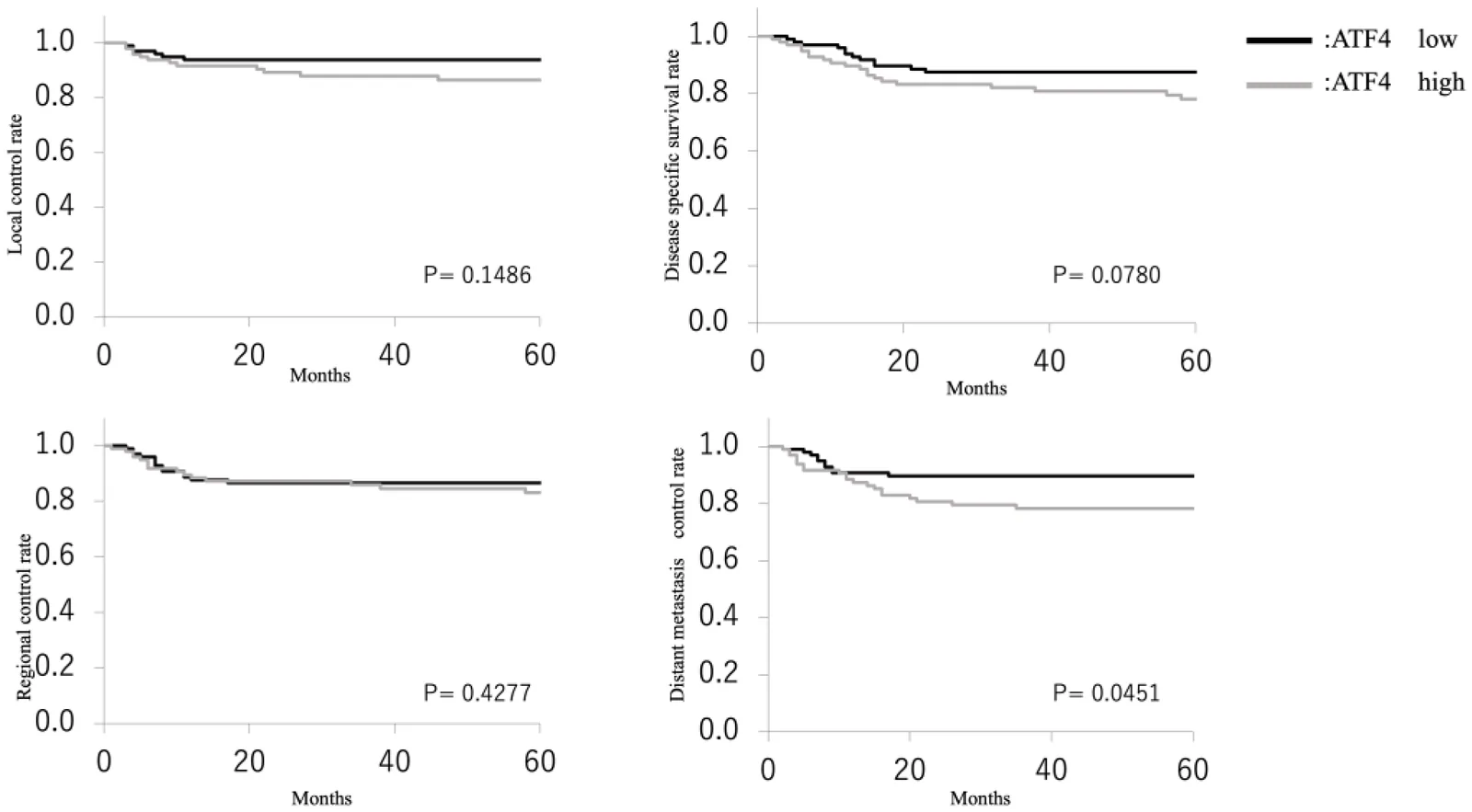
The DM control rate was significantly worse in the high ATF4 expression group than in the low expression group (*p* = 0.045). In contrast, no significant differences were observed in the LC rate (*p* = 0.149), DSS (*p* = 0.078), or RC rate (*p* = 0.428).

Kaplan–Meier survival analysis evaluated associations between expression levels of NRF2, TFEB, Parkin, PINK1, MITOL, and ATF4 (high-expression group vs. low-expression group) and prognosis over a 60-month follow-up period (Figure 2, Figure 3, Figure 4, Figure 5, Figure 6 and Figure 7). Endpoints included LC, DSS, RC, and DM control.

Explanatory multivariable analysis revealed associations of high TFEB expression (HR = 4.085), vascular invasion (HR = 3.038), and ENE (HR = 3.145) with disease-specific mortality (*p* = 0.012, *p* = 0.021, and *p* = 0.009, respectively) (Table 4).

Furthermore, the results of the explanatory multivariate analysis with the 11 factors were consistent with the present findings.

## 4. Discussion

Mitophagy is a selective form of autophagy that eliminates damaged mitochondria and is essential for maintaining mitochondrial quality control [1,2]. In this study, we comprehensively analyzed the expression of mitophagy-associated factors in OSCC and examined their association with clinicopathological parameters and patient outcomes. High expression of TFEB, NRF2, and ATF4 was related with poor clinical outcomes, and an explanatory multivariable analysis showed an association between high TFEB expression and disease-specific mortality. In contrast, no clear prognostic association was observed for the core components of the PINK1–Parkin pathway, including Parkin, PINK1, and MITOL. These findings suggest that mitophagy regulation in OSCC may not rely on a single pathway and may instead be governed by higher-order transcriptional networks, including the coordinated lysosomal expression and regulation (CLEAR) network, which integrates multiple stress-adaptive mechanisms.

The role of mitophagy in cancer varies according to the tumor development stage. During the “true early phase” of tumorigenesis, when normal cells initially undergo malignant transformation, mitophagy is considered to suppress tumor formation by maintaining mitochondrial integrity. In contrast, once a tumor has been established—even in the “early cancer” stage—mitophagy may contribute to tumor cell survival and progression by enhancing stress resistance and metabolic adaptation. In this study, NRF2 was more frequently negative in advanced tumors (T3/T4) (*p* = 0.015). However, cases with high NRF2 expression showed a worse prognosis in Kaplan–Meier analysis. This observation suggests that NRF2 expression may be related with the aggressiveness of tumors rather than with their T classification.

Two major mitophagy pathways have been established: the PINK1–Parkin pathway and receptor-mediated mitophagy involving BNIP3, NIX, and FUNDC1 [12]. The PINK1–Parkin pathway represents an acute damage–response mechanism activated by loss of mitochondrial membrane potential, and appropriate expression of these molecules exerts tumor-suppressive roles in various cancer types. In early tumorigenesis and non-advanced cancers, PINK1–Parkin-dependent mitophagy has been reported to suppress ROS accumulation and maintain genome stability, thereby preventing tumor formation [1,13]. In contrast, their prognostic significance in advanced cancers or under chronic stress conditions remains inconsistent and appears to depend on tumor stage and treatment context [14].

MITOL, an E3 ubiquitin ligase localized to the mitochondrial outer membrane, regulates mitochondrial dynamics and quality control [5]. During mitophagy, MITOL promotes the degradation of phosphorylated Parkin, thereby modulating Parkin activity and preventing excessive mitophagy while stabilizing the anti-apoptotic protein FKBP38 [5]. These findings indicate that MITOL functions as a regulatory modulator that fine-tunes mitophagy activity rather than a molecule that directly drives the PINK1–Parkin pathway. Therefore, MITOL is more likely to be involved in maintaining mitochondrial homeostasis under cellular stress conditions than in directly determining tumor aggressiveness or prognosis. Only limited studies have reported direct evaluations of the prognostic significance of Parkin, PINK1, and MITOL in OSCC or head and neck squamous cell carcinoma (HNSCC), and their clinical implications remain inconclusive [15].

ATF4 and NRF2 are stress-responsive transcription factors that induce multiple mitophagy-related genes, including PINK1 and Parkin [6,8,16]. However, their roles in OSCC are not necessarily identical, and distinct characteristics have been reported.

NRF2 activation contributes to increased antioxidant capacity, treatment resistance, tumor progression, and poor prognosis [6,7,15]. In OSCC, NRF2 activation has been related with high aggressiveness and unfavorable clinical outcomes. Yamadori et al. reported that NRF2 promotes tumor growth in non-small-cell lung cancer [17], and similar proliferation-enhancing effects are believed to occur in OSCC. Matsuoka et al. demonstrated that nuclear accumulation of NRF2 was significantly related with radioresistance and poor distant control in OSCC [18]. Furthermore, Namani et al. identified a *KEAP1*–*NRF2*–*CUL3* regulatory gene signature strongly related with poor prognosis in HNSCC [19]. These findings align with current results showing that high NRF2 expression was related with tumor aggressiveness.

In contrast, few clinical studies have reported direct examinations of the prognostic relevance of ATF4 in OSCC. Although numerous basic studies have reported that ATF4 contributes to cell survival, invasion, and metabolic adaptation [8,15,20], clinical evidence evaluating ATF4 expression by immunohistochemistry and examining associations with DSS remains scarce. In this study, high ATF4 expression demonstrated an association with poor DM control, providing novel insight into the clinical significance of ATF4. Notably, among the six factors evaluated, only ATF4 showed a significant association with close surgical margins. Previous studies have reported an association between ATF4 and high aggressiveness [21,22]; the present findings support the clinical validity of ATF4 as a marker of tumor aggressiveness. Tumors with high ATF4 expression may remain close to the surgical margin despite attempts to secure adequate margins, indicating a strong influence on invasive potential compared to other mitophagy-associate factors.

Moreover, NRF2 and ATF4 represent functionally interacting pathways rather than independent mechanisms. NRF2 indirectly regulates ATF4 activity via amino acid metabolism-related factors, and the cooperation between both controls metabolic pathways such as serine biosynthesis, thereby enhancing stress adaptation in tumor cells [23]. ATF4 induces the expression of Parkin and BNIP3 under stress conditions [16,20], whereas NRF2 directly regulates PINK1 transcription [6,24]. Thus, NRF2 and ATF4 act in complementary and cooperative manners within mitophagy regulation.

**Figure 8:**
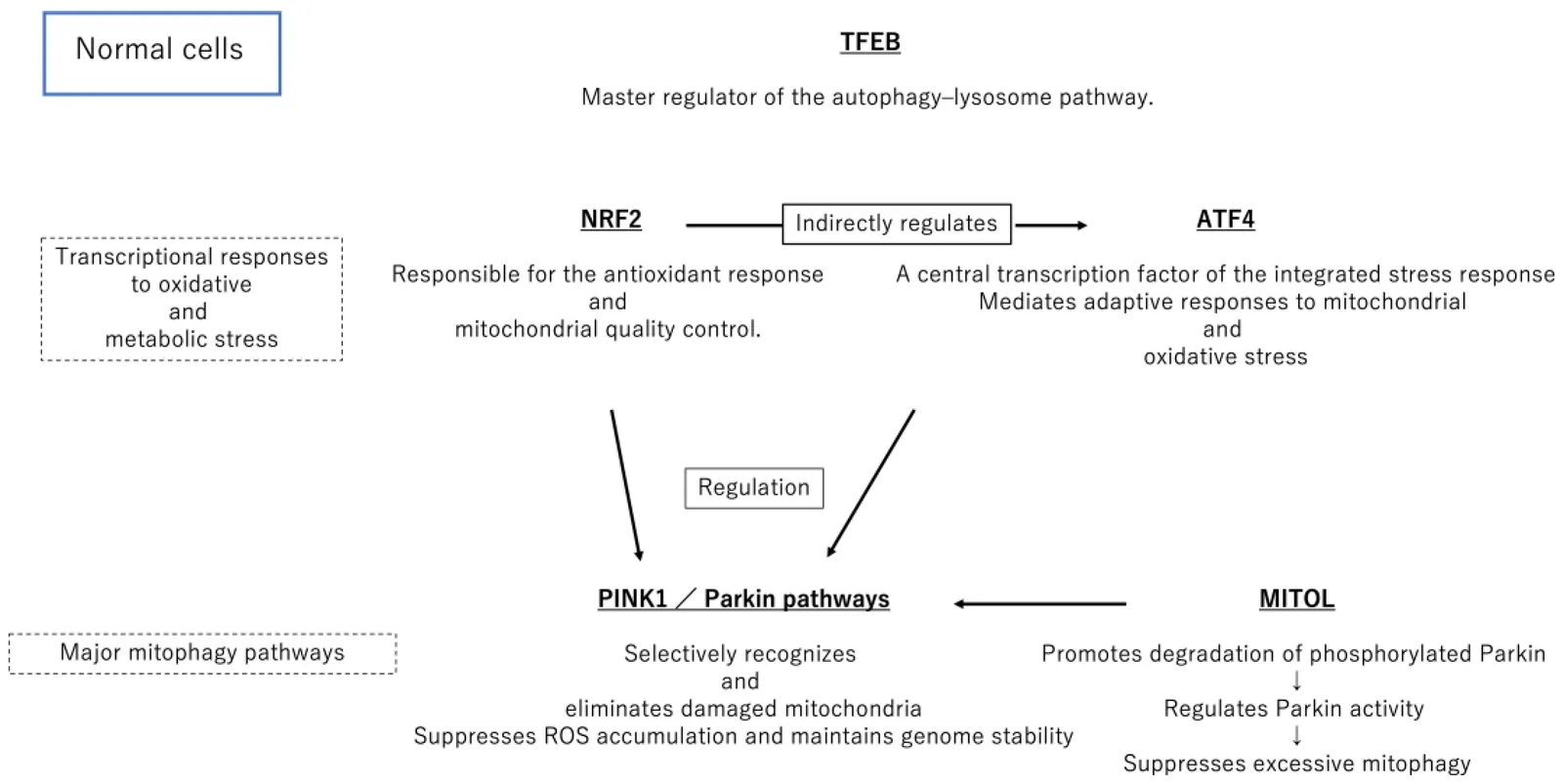
Schematic representation of mitophagy-associated factors and their interactions in normal cells.

By contrast, TFEB regulates autophagy–lysosome pathways at a hierarchical level distinct from the previously discussed factors. TFEB functions as a master transcription factor controlling lysosomal biogenesis and autophagy-related gene expression through the CLEAR network (Figure 8) [4]. Constitutive TFEB activation has been reported in multiple malignancies, such as pancreatic, hepatic, melanoma, and lung cancers, and is associated with metabolic reprogramming, therapeutic resistance, and enhanced invasion and metastasis [3,24,25,26]. High TFEB expression or nuclear localization has also been reported as a poor prognostic factor in multiple cancers, including oral cancer [8,27,28]. Sakamoto et al. showed that TFEB knockdown reduced invasion and migration in OSCC cells [27], whereas Tsai et al. demonstrated that TFEB nuclear translocation promotes lysosome-related gene expression and affects drug sensitivity in oral cancer cells [29]. These reports align with the current findings showing that high TFEB expression was strongly related with decreased DSS, poor RC, and DM control.

TFEB acts as a context-dependent factor, exerting different roles in normal versus tumor cells. In tumors, TFEB may repurpose intracellular quality control systems, including mitophagy, as survival strategies. Recent studies have also reported that TFEB regulates PINK1–Parkin-independent mitophagy, particularly BNIP3/NIX-dependent pathways under hypoxic conditions [12,30,31]. Thus, TFEB does not directly control PINK1 or Parkin and activates the autophagy–lysosome machinery as a whole, creating a cellular environment that permits both PINK1–Parkin-dependent and -independent mitophagy [4].

OSCC is frequently exposed to chronic hypoxia, oxidative stress, and nutrient deprivation within the tumor microenvironment. Under such conditions, the autophagy–lysosome system may contribute to tumor-cell adaptation and survival. These tumor microenvironment characteristics may partly explain why TFEB, rather than PINK1 or Parkin, showed an association with prognosis. Furthermore, the stronger prognostic association observed for TFEB compared with NRF2 and ATF4 may reflect differences in the biological functions and cellular contexts represented by these stress-responsive factors.

In an explanatory multivariable analysis, TFEB was also related with DSS. This finding further supports the potential prognostic relevance of TFEB expression; however, it does not establish a direct functional role of TFEB in mitophagy or tumor progression.

This study had some limitations. First, as a retrospective study, the influence of treatment-related and unmeasured confounding factors could not be fully excluded despite performing explanatory multivariable analysis. For example, postoperative adjuvant treatments, including radiotherapy and chemoradiotherapy, were not included as variables in the present analysis and may represent potential confounding factors. Second, immunohistochemical evaluation measures steady-state protein abundance and does not directly assess mitophagy flux or mitochondrial turnover. In addition, TFEB, NRF2, and ATF4 have biological functions beyond mitophagy. Therefore, the present findings should be interpreted as associations between the expression of mitophagy-associated factors and clinicopathological characteristics or patient outcomes rather than as direct evidence of altered mitophagy activity. Third, dedicated positive control tissues and antibody validation using knockdown or knockout approaches were not included in the present study, which represents a limitation of the immunohistochemical evaluation. Fourth, cell counts were determined by consensus between two observers rather than through independent assessments; therefore, inter-observer reproducibility could not be formally evaluated. Future studies should include independent assessments by multiple observers with appropriate statistical evaluation of reproducibility. Fifth, the use of median values as cutoffs may have resulted in loss of information and may not represent biologically or clinically optimal thresholds. Sixth, although TFEB expression remained related with DSS in the explanatory multivariable analysis, the analysis was based on only 33 DSS events. Therefore, the statistical robustness of this finding is limited, and the association between TFEB expression and prognosis should be interpreted cautiously rather than as evidence of a strong or direct prognostic relationship.

## 5. Conclusions

We investigated the expression of mitophagy-associated factors (TFEB, NRF2, ATF4, Parkin, PINK1, and MITOL) and their association with prognosis in patients with OSCC. High expression of NRF2 and TFEB was significantly related with poor prognosis, whereas ATF4 expression was related with DM control. Among these factors, TFEB showed an exploratory association with disease-specific mortality in the explanatory multivariable analysis. In contrast, Parkin, PINK1, and MITOL showed no clear association with prognosis. These findings indicate that stress-responsive transcriptional regulators such as NRF2, TFEB, and ATF4 may be related with tumor aggressiveness and prognosis in OSCC, whereas individual components of the PINK1–Parkin pathway did not show clear prognostic associations in this cohort. Because, in the present study, we assessed protein expression by immunohistochemistry rather than mitophagy flux or mitochondrial turnover, the findings do not support altered mitophagy activity. Further studies are required to validate these findings, clarify their biological mechanisms, and determine their potential clinical utility as prognostic biomarkers in OSCC.

## Figures and Tables

**Table 1:** Cutoff values for positive expression of each factor.

Factor	Cutoff Value (Median), %	Negative (Low Expression)	Positive (High Expression)
NRF2	47.57	≤47.57%	>47.57%
TFEB	38.20	≤38.20%	>38.20%
Parkin	51.54	≤51.54%	>51.54%
PINK1	50.13	≤50.13%	>50.13%
MITOL	38.71	≤38.71%	>38.71%
ATF4	52.84	≤52.84%	>52.84%

The cutoff value for each factor was defined as the cohort-specific median of the positive-cell ratio (percentage of immunopositive tumor cells) among all 197 patients. Cases with expression levels greater than the median were classified as positive (high expression); cases with expression levels equal to or below the median were classified as negative (low expression).

**Table 2:** Clinicopathological characteristics of patients with OSCC.

Characteristics of Patients	*n*	(%)
Age		
<70	100	50.8
≥70	97	49.2
Sex		
Male	106	53.8
Female	91	46.2
PS		
0 or 1	190	96.4
More than 2	7	3.6
Exposure to tobacco		
Smoker	59	29.9
Non	138	70.1
Exposure to alcohol		
Drinker	77	39.1
Non	120	60.9
Primary tumor site		
Tongue	92	46.7
Lower gingiva	43	21.9
Upper gingiva	30	15.2
Buccal mucosa	16	8.1
Oral floor	16	8.1
Palate	0	0
Clinical T stage		
1	36	18.3
2	66	33.5
3	30	15.2
4a/b	65	33.0
Clinical N stage		
0 or 1	154	78.2
2 or 3	43	21.8
Lymphatic invasion		
Positive	41	20.8
Negative	156	79.2
Vascular invasion		
Positive	61	31.0
Negative	136	69.0
Nerve invasion		
Positive	41	20.8
Negative	156	79.2
Tumor differentiation		
Well	109	55.4
Moderate	71	36.0
Poor	17	8.6
Margin		
Positive	28	14.2
Negative	169	85.8
Multiple neck metastases		
Positive	37	18.8
Negative	87	44.2
Non-neck dissection	73	37.0
ENE		
Positive	25	12.7
Negative	42	21.3
Non-lymph node metastasis; non-neck dissection	130	66.0
Disease control Status		
Survival	143	72.6
Local recurrence	20	10.2
Regional recurrence	32	16.2
Distant metastasis	32	16.2
Disease-specific death	33	16.8
Other causes	21	10.7

ENE: extranodal extension.

**Table 3:** Associations between the protein expressions of mitophagy-associated factors and clinicopathological parameters.

			**NRF2**			**TFEB**			**ATF4**		
		***n***	**Low** **Expression**	**High** **Expression**	***p*** **-Value**	**Low** **Expression**	**High** **Expression**	***p*** **-Value**	**Low** **Expression**	**High** **Expression**	***p*** **-Value**
Age	<70	100	49	51	0.776	52	48	0.670	53	47	0.477
	≥70	97	50	47		47	50		46	51	
Sex	Male	106	54	52	0.887	48	58	0.154	60	46	0.064
	Female	91	45	46		51	40		39	52	
PS	0 or 1	190	95	95	1.000	95	95	1.000	95	95	1.000
	More than 2	7	4	3		4	3		4	3	
Exposure to tobacco	Smoker	59	30	29	1.000	31	28	0.756	31	28	0.756
	Non	138	69	69		68	70		68	70	
Exposure to alcohol	Drinker	77	42	35	0.382	38	39	0.884	40	37	0.771
	Non	120	57	63		61	59		59	61	
Primary tumor site	Tongue	92	47	45	0.887	41	51	0.154	49	43	0.476
	Otherwise	105	52	53		58	47		50	55	
Clinical T stage	T1, T2	102	60	42	0.015	57	45	0.118	55	47	0.320
	T3, T4	95	39	56		42	53		44	51	
Clinical N stage	0 or 1	154	83	71	0.059	83	71	0.059	76	78	0.731
	>2	43	16	27		16	27		23	20	
Lymphatic invasion	Positive	41	18	23	0.385	19	22	0.602	24	17	0.293
	Negative	156	81	75		80	76		75	81	
Vascular invasion	Positive	61	28	33	0.444	30	31	0.878	29	32	0.646
	Negative	136	71	65		69	67		70	66	
Nerve invasion	Positive	41	21	20	1.000	17	24	0.223	24	17	0.293
	Negative	156	78	78		82	74		75	81	
Tumor differentiation (Grade2)	Well	109	56	53	0.775	49	60	0.115	52	57	0.475
	Moderate–poorly	88	43	45		50	38		47	41	
Margin	Close to margin–positive	28	11	17	0.227	16	12	0.541	9	19	0.043
	Negative	169	88	81		83	86		90	79	
Multiple neck metastases	Positive	37	12	25	0.052	15	22	0.555	14	23	0.239
	Negative	87	45	42		42	45		44	43	
ENE	Positive	25	9	16	0.609	10	15	0.800	9	16	0.609
	Negative	42	19	23		19	23		19	23	
			**Parkin**			**PINK1**			**MITOL**		
		***n***	**Low** **Expression**	**High** **Expression**	***p*** **-Value**	**Low** **Expression**	**High** **Expression**	***p*** **-Value**	**Low** **Expression**	**High** **Expression**	***p*** **-Value**
Age	<70	100	47	53	0.394	47	53	0.394	50	50	1.000
	≥70	97	52	45		52	45		49	48	
Sex	Male	106	58	48	0.200	54	52	0.887	53	53	1.000
	Female	91	41	50		45	46		46	45	
PS	0 or 1	190	94	96	0.445	96	94	0.721	96	94	0.721
	More than 2	7	5	2		3	4		3	4	
Exposure to tobacco	Smoker	59	32	27	0.534	31	28	0.756	31	28	0.756
	Non	138	67	71		68	70		68	70	
Exposure to alcohol	Drinker	77	37	40	0.663	41	36	0.560	38	39	0.884
	Non	120	62	58		58	62		61	59	
Primary tumor site	Tongue	92	45	47	0.776	42	50	0.255	34	58	0.001
	Otherwise	105	54	51		57	48		65	40	
Clinical T stage	T1, T2	102	58	44	0.064	53	49	0.670	52	50	0.887
	T3, T4	95	41	54		46	49		47	48	
Clinical N stage	0 or 1	154	80	74	0.393	76	78	0.731	83	71	0.059
	>2	43	19	24		23	20		16	27	
Lymphatic invasion	Positive	41	21	20	1.000	21	20	1.000	18	23	0.385
	Negative	156	78	78		78	78		81	75	
Vascular invasion	Positive	61	30	31	0.878	31	30	1.000	32	29	0.758
	Negative	136	69	67		68	68		67	69	
Nerve invasion	Positive	41	17	24	0.223	22	19	0.726	17	24	0.223
	Negative	156	82	74		77	79		82	74	
Tumor differentiation (Grade2)	Well	109	51	58	0.317	50	59	0.198	54	55	0.886
	Moderate–poorly	88	48	40		49	39		45	43	
Margin	Close to margin–positive	28	13	15	0.688	10	18	0.107	11	17	0.227
	Negative	169	86	83		89	80		88	81	
Multiple neck metastases	Positive	37	18	19	0.558	18	19	0.845	15	22	0.555
	Negative	87	37	50		45	42		42	45	
ENE	Positive	25	13	12	0.613	12	13	0.803	8	17	0.131
	Negative	42	18	24		22	20		22	20	

**Table 4:** Relationship between operative variables, including the protein expressions of mitophagy-associated factors, and DSS in the explanatory multivariable analysis.

Variable	*p*-Value	Hazard Ratio	95% CI	
			Lower	Upper
High TFEB expression	0.012	4.085	1.368	12.202
Vascular invasion	0.021	3.038	1.180	7.819
Extranodal extension	0.009	3.145	1.337	7.396

Statistically significant (*p* < 0.05). CI: Confidence interval.

## Data Availability

The data presented in this study are available on request from the corresponding author. (The data are not publicly available due to privacy or ethical restrictions).

## Abbreviations

The following abbreviations are used in this manuscript:

ATF4	Activating transcription factor 4
CI	Confidence interval
CLEAR	Coordinated lysosomal expression and regulation
DM	Distant metastasis
DSS	Disease-specific survival
ENE	Extranodal extension
HNSCC	Head and neck squamous cell carcinoma
HR	Hazard ratio
LC	Local control
MARCH5	Membrane-associated ring-CH-type finger 5
MITOL	Mitochondrial ubiquitin ligase
MLM	Multiple lymph node metastasis
NRF2	Nuclear factor erythroid 2-related factor 2
OSCC	Oral squamous cell carcinoma
PINK1	PTEN-induced kinase 1
PS	Performance status
RC	Regional control
ROS	Reactive oxygen species
TFEB	Transcription factor EB
